# Virologic Response to Very Early HIV Treatment in Neonates

**DOI:** 10.3390/jcm10102074

**Published:** 2021-05-12

**Authors:** Stephanie Shiau, Renate Strehlau, Yanhan Shen, Yun He, Faeezah Patel, Megan Burke, Elaine J. Abrams, Caroline T. Tiemessen, Shuang Wang, Louise Kuhn

**Affiliations:** 1 Department of Biostatistics and Epidemiology, Rutgers School of Public Health, Piscataway, NJ 08854, USA; 2 Empilweni Services and Research Unit, Rahima Moosa Mother and Child Hospital, Department of Paediatrics and Child Health, Faculty of Health Sciences, University of the Witwatersrand, Johannesburg 2112, South Africa; Renate.Strehlau@wits.ac.za (R.S.); FPatel@wrhi.ac.za (F.P.); megan.burke08@gmail.com (M.B.); 3 Gertrude H. Sergievsky Center, Vagelos College of Physicians and Surgeons, Columbia University, New York, NY 10032, USA; ys2909@cumc.columbia.edu (Y.S.); lk24@cumc.columbia.edu (L.K.); 4 Department of Biostatistics, Mailman School of Public Health, Columbia University, New York, NY 10032, USA; yh3094@cumc.columbia.edu (Y.H.); sw2206@cumc.columbia.edu (S.W.); 5 Department of Epidemiology, Mailman School of Public Health, Columbia University, New York, NY 10032, USA; eja1@cumc.columbia.edu; 6 ICAP at Columbia University, Mailman School of Public Health, Columbia University, New York, NY 10032, USA; 7 Department of Pediatrics, Vagelos College of Physicians & Surgeons, Columbia University, New York, NY 10032, USA; 8 Centre for HIV and STIs, National Institute for Communicable Diseases, National Health Laboratory Services, and Faculty of Health Sciences, University of the Witwatersrand, Johannesburg 2193, South Africa; carolinet@nicd.ac.za

**Keywords:** viral suppression, viral response, ARV, neonates, infants, pediatrics

## Abstract

Factors that influence viral response when antiretroviral therapy (ART) is initiated in neonates are not well characterized. We assessed if there is consistency in predictive factors when operationalizing viral response using different methods. Data were collected from a clinical study in South Africa that started ART in neonates within 14 days of birth (2013–2018). Among 61 infants followed for ≥48 weeks after ART initiation, viral response through 72 weeks was defined by three methods: (1) clinical endpoints (virologic success, rebound, and failure); (2) time to viral suppression, i.e., any viral load (VL: copies/mL) <400, <50, or target not detected (TND) using time-to-event methods; and (3) latent class growth analysis (LCGA) to empirically estimate discrete groups with shared patterns of VL trajectories over time. We investigated the following factors: age at ART initiation, sex, birthweight, preterm birth, mode of delivery, breastfeeding, pre-treatment VL and CD4, maternal ART during pregnancy, and maternal VL and CD4 count. ART was initiated 0–48 h of birth among 57.4% of the infants, 48 h–7 days in 29.5% and 8–14 days in 13.1%. By Method 1, infants were categorized into ‘success’ (54.1%), ‘rebound’ (21.3%), and ‘failure’ (24.6%) for viral response. For Method 2, median time to achieving a VL <400, <50, or TND was 58, 123, and 331 days, respectively. For Method 3, infants were categorized into three trajectories: ‘rapid decline’ (29.5%), ‘slow decline’ (47.5%), and ‘persistently high’ (23.0%). All methods found that higher pre-treatment VL, particularly >100,000, was associated with less favorable viral outcomes. No exposure to maternal ART was associated with a better viral response, while a higher maternal VL was associated with less favorable viral response and higher maternal CD4 was associated with better viral response across all three methods. The LCGA method found that infants who initiated ART 8–14 days had less favorable viral response than those who initiated ART earlier. The other two methods trended in a similar direction. Across three methods to operationalize viral response in the context of early infant treatment, findings of factors associated with viral response were largely consistent, including infant pre-treatment VL, maternal VL, and maternal CD4 count.

## 1. Introduction

The 2013 report of the Mississippi baby who started treatment <30 h of birth and maintained viral suppression off treatment for over two years raised the possibility that very early initiation of antiretroviral therapy (ART) close to birth could lead to better virologic outcomes, as well as periods of ART-free HIV remission in other infants [1,2]. The benefits of earlier ART initiation on long-term virologic control have been reported in studies of infants [3,4,5,6,7]. However, little data are available on viral response in those initiating ART very early in the neonatal period.

We conducted a study in South Africa to test whether very early initiation of ART would allow a sizable proportion of infants to meet protocol-specified virologic and immunologic endpoints in order to justify an interruption trial to attain ART-free HIV remission. We found that the proportion of infants meeting the virologic endpoint was similar in those who started ART <48 h and those who started between 2–14 days [8]. This finding appeared to contradict existing evidence of a benefit of early treatment on viral suppression, but most other studies included infants initiating ART up to 3–6 months of age rather than the first two weeks of life [3]. Although resource intensive and logistically challenging, initiation of ART <48 h is possible with rapid diagnostic testing and turnaround. Thus, it would be helpful to know whether very early treatment has measurable benefits on long-term virologic outcomes.

Choice of virologic endpoint is also important to consider. Historically, studies of HIV treatment have used different virologic endpoints as the primary indicator of treatment success [9,10,11]. In contrast to our study, a pooled analysis found that infants initiated on ART <7 days were more likely to achieve viral suppression, defined as ≥2 consecutive measurements ≤50 copies/mL, than those initiated on ART 8–28 days using a time-to-event-analysis [12]. This virologic endpoint was defined differently than the one used in our study.

Therefore, in this study, we posited it would be informative to operationalize viral response using different methods, and aimed to determine whether age at ART initiation and other factors would consistently be associated with these different conceptualizations of viral endpoints.

## 2. Materials and Methods

### 2.1. Study Population

A clinical study of very early treatment was conducted at Rahima Moosa Mother and Child Hospital in Johannesburg, South Africa to test whether a sizable minority of HIV-infected neonates started on ART <14 days of birth and maintained on ART for at least two years would be able to control viremia when ART was withdrawn (2013–2018) [8]. As part of the protocol, 73 intrauterine HIV-infected neonates identified <48 h of birth were initiated on ART <14 days of birth. The initial ART regimen consisted of nevirapine, lamivudine and zidovudine if the infant was <42 weeks post-menstrual age. Nevirapine was changed to lopinavir-ritonavir no sooner than 42 weeks post-menstrual age accounting for patient readiness. For this analysis, 61 neonates who survived and were followed for ≥48 weeks were included (3 died, 9 not followed ≥48 weeks). Protocols were approved by the Institutional Review Boards of Columbia University (IRB-AAAO5011; approved 15 December 2014) and the University of the Witwatersrand (M141029; approved 31 October 2014). Mothers signed informed consent for their own and their infant’s participation.

### 2.2. Measurements

Quantitative HIV-1 RNA in plasma [viral load (VL)] was measured for the infants pre-treatment and 1, 2, 4, 8, 12, 16, 20, 24, 32, 40, 48, 60, and 72 weeks of age (COBAS AmpliPrep/COBAS TaqMan HIV-1 test, v2.0, Roche Molecular Systems, Inc., Branchburg, NJ, USA). Maternal VLs were measured during late pregnancy, at delivery, or soon thereafter using the same assay. Infant pre-treatment CD4^+^ T-cell count and percentages and maternal CD4^+^ T-cell count and percentages close to delivery were measured by the TruCount Method (BD Biosciences, Heidelberg, Germany). Antenatal and birth characteristics were also collected, including maternal age, maternal ART during pregnancy, infant sex, birthweight, gestational age, mode of delivery, age at ART initiation, and breastfeeding status.

### 2.3. Statistical Analysis

This analysis aimed to describe viral response through 72 weeks using different methods, compare the methods, and determine if there was consistency in factors associated with viral response across the methods. Three methods were used to define viral response: (1) clinical endpoints, (2) time to viral suppression, and (3) latent class growth analysis (LCGA) (Supplementary Table S1):

The first method categorized infants into three viral response groups based on clinically meaningful viral endpoints (virologic success, rebound, and failure) through 72 weeks. In our trial protocol, the primary virologic endpoint was a VL <400 copies/mL by 24 weeks after ART initiation and a VL <50 copies/mL by 48 weeks of age, and no confirmed VL (i.e., two consecutive) >50 copies/mL after suppression was attained. For this analysis, we used this endpoint to define virologic success through 72 weeks. Virologic rebound was defined as having a VL <400 copies/mL by 24 weeks after ART initiation, a VL <50 copies/mL by 48 weeks of age, and confirmed VL >50 copies/mL after suppression was attained through 72 weeks. Viral failure was defined as never having achieved VL <400 copies/mL by 48 weeks of age [8]. Factors of interest were compared across groups using ANOVA and Wilcoxon tests for continuous variables and Chi-squared or Fisher’s exact tests for categorical variables. Trend tests were performed [13].

The second method defined the virologic endpoint as time to any VL (copies/mL) <400, <50, or target not detected (TND) by 72 weeks. Kaplan–Meier methods were used to plot survival curves. Follow-up time for the children was censored at their last follow-up visit. Associations between factors of interest and viral suppression were assessed using Cox proportional hazards models to estimate hazard ratios and 95% confidence intervals (95% CI).

The third method used latent class growth analysis (LCGA) to empirically estimate discrete groups of infants with shared patterns of VL trajectories over time. Briefly, LCGA is a method for clustering individuals with similar patterns of a characteristic of interest over time [14,15]. We fit latent trajectories on log10-transformed VLs (copies/mL) over time and selected the best fitting model with number of latent groups using the Bayesian Information Criterion (BIC), and the group membership posterior probability. The final model was selected based on (1) the inflection point at which the ΔBIC leveled off [14,15,16], and (2) clinical meaning of each group. Trajectory membership was treated as the outcome variable. Factors of interest were compared across groups using ANOVA and Wilcoxon tests for continuous variables and Chi-squared or Fisher’s exact tests for categorical variables. Trend tests were performed.

The primary factor of interest was age at ART initiation assessed as both a continuous variable (days) and a categorical variable in 3 categories: 0–48 h, >48 h–7 days, 8–14 days. Other factors investigated were: infant sex, birthweight, preterm birth, mode of delivery, breastfeeding status, infant pre-treatment VL, and CD4 percentage and count, maternal ART during pregnancy, and maternal VL and CD4 count closest to birth. For infants missing pre-treatment CD4, we conducted a sensitivity analysis imputing the CD4 value based on the observed pre-treatment VL and a linear regression model from those with both measures available. Analyses were conducted using SAS software version 9.4 (Cary, NC, USA).

## 3. Results

Characteristics of the 61 mother-infant pairs are presented in Table 1. A little over half (57.4%) of the infants initiated ART at 0–48 h, 29.5% at 48 h–7 days and 13.1% at 8–14 days. Of the 61 infants, 50.8% were male, mean birthweight was 2766 ± 629 (range 905–4150 g), and 16.4% were born pre-term. Approximately three-quarters of the infants were born via vaginal delivery. Mean maternal age was 28.8 ± 6.1 years; 16.4% started ART before pregnancy, 65.6% started ART while pregnant, and 18% did not receive any ART until delivery. Infant pre-treatment VL was available for 93.4% of infants and measured at a median of 1 (IQR: 1–6 days). Median pre-treatment VL was 19,400 (IQR: 1720–240,810 copies/mL). Infant pre-treatment CD4 measurements were available for 82.0% of infants. Mean CD4 count was 1990 ± 995 cells/mm^3^ and CD4 percentage was 41.4 ± 13.2%. 

**Figure 1:**
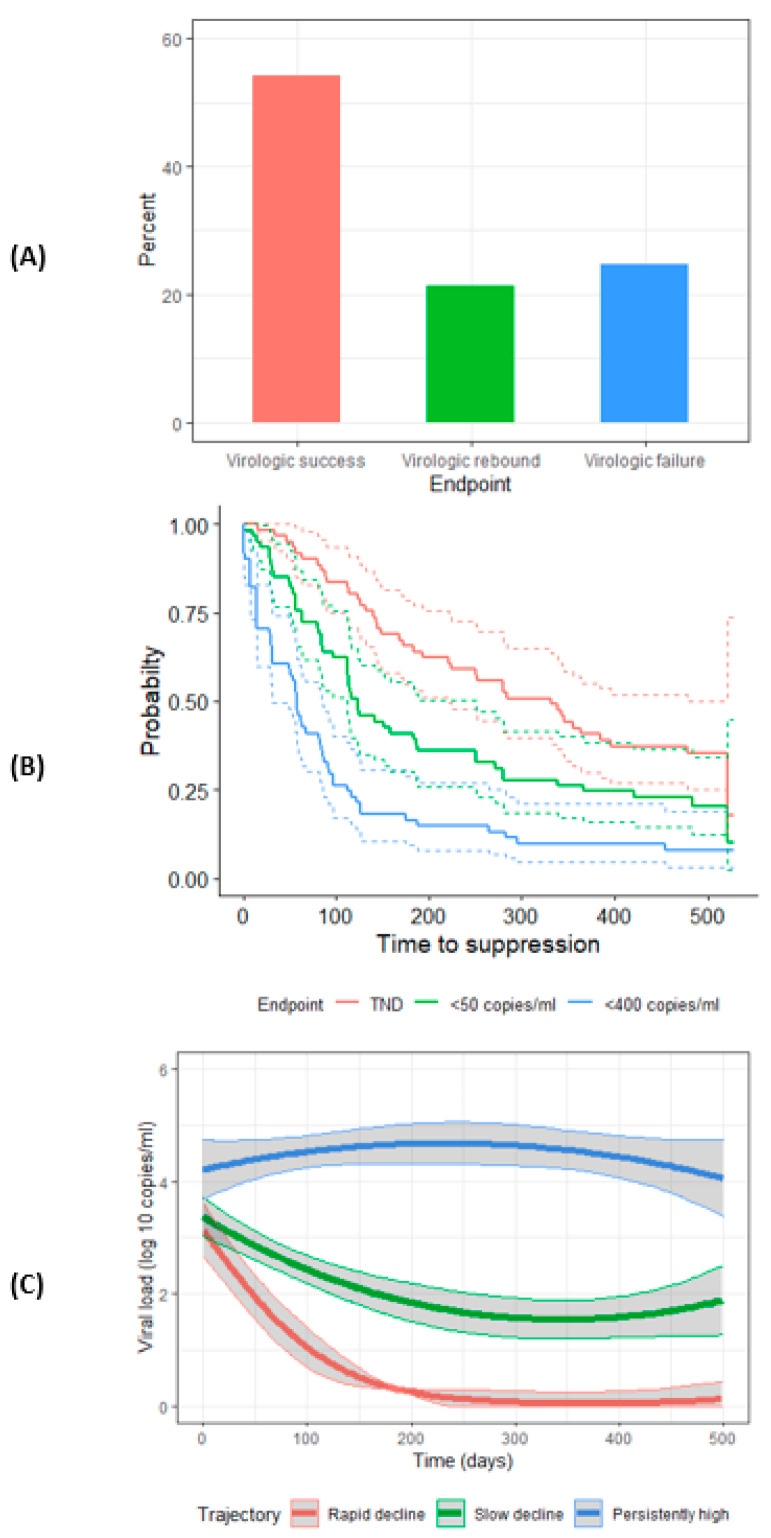
Pattern of viral response through 72 weeks for 61 intrauterine HIV-infected neonates by three methods. (**A**) Bar graph depicting percentage of participants with each clinically-meaningful viral load endpoint (virologic success, rebound, and failure) through 72 weeks. (**B**) Kaplan–Meier curves depicting probability of achieving a viral load <400 copies/mL, <50 copies/mL, and target not detected (TND) by 72 weeks; dotted lines represent 95% confidence intervals. (**C**) Trajectories of viral load (log10 copies/mL) through 72 weeks of age as determined by latent class growth model; bands represent 95% confidence intervals.

Viral response through 72 weeks using the three methods is described in Figure 1. For the first method which categorized infants into viral response groups based on clinically meaningful viral endpoints through 72 weeks, 33 (54.1%) infants were in the ‘virologic success’ group, 13 (21.3%) in the ‘virologic rebound’ group, and 15 (24.6%) in the ‘virologic failure’ group (Figure 1A). 

For the second method, Kaplan–Meier probabilities of achieving a VL <400 copies/mL, <50 copies/mL, or TND by 72 weeks are shown in Figure 1B. The median time to achieving a VL <400 copies/mL, <50 copies/mL, or TND was 58 days (95% CI: 31–83), 123 days (95% CI: 97–186), and 331 days (95% CI: 194–396), respectively. 

Finally, for the third method, the best fitting LCGA model based on ΔBIC classified the children into three trajectories of VL (Supplementary Table S2). Of the three trajectories, one was characterized by VL dropping fast and remaining low (‘rapid decline’). The second was defined by VL dropping slowly (‘slow decline’). The third was defined by VL remaining persistently high through 72 weeks (‘persistently high’) (Figure 1C). Eighteen (29.5%), 29 (47.5%), and 14 (23.0%) of children were assigned to ‘rapid decline’, ‘slow decline’, and ‘persistently high’, respectively.

Some but not all infants were categorized into the same groups by Methods 1 and 3. All 18 infants in the ‘rapid decline’ trajectory (Method 3) were in the ‘virologic success’ group defined by the clinical endpoints (Method 1). Of the 29 in the ‘slow decline’ trajectory, 14 (48.3%) were in the ‘virologic success’ group, 11 (37.9%) were in the ‘virologic rebound’ group and 4 (13.8%) were in the ‘virologic failure’ group. Of the 14 infants in the ‘persistently high’ trajectory, most (78.6%) were in the ‘virologic failure’ group, while 2 (14.3%) and 1 (7.1%) were in the ‘virologic rebound’ and ‘virologic success’ groups, respectively.

Next, we examined if there was consistency in factors associated with viral response across the methods (Table 2, Table 3 and Table 4). We found that infant age at ART initiation was not significantly associated with viral response using the clinical endpoints, but trended in the expected direction, with an older mean age in the ‘virologic failure’ group (4.9 days) than the ‘virologic success’ and ‘virologic rebound’ groups (2.9 and 2.5 days, respectively) (Table 2). In addition, a greater proportion of infants in the ‘virologic failure’ group (26.7%) initiated ART 8–14 days compared to the other groups (9.1% and 7.7%). Using the second method, when compared to ART initiation 8–14 days, earlier ART initiation 0–48 h (HR: 2.46, 95% CI: 0.94–6.45) and >48 h–7 days (HR: 2.18, 95% CI: 0.89–6.03) trended towards a higher probability of suppression <50 copies/mL (Table 3).

The trajectory membership group approach found a significant association between age at ART initiation and viral response; infants in the ‘persistently high’ trajectory initiated ART at an older age (6.0 days) than the ‘rapid decline’ and ‘slow decline’ groups (2.7 and 2.4 days, respectively) (*p* = 0.008) (Table 4). This was also reflected categorically, with a higher proportion of infants with a ‘persistently high’ trajectory (35.7%) initiating ART 8–14 days compared to the other two groups (*p* = 0.03).

Across all three methods, infant sex, birthweight, preterm birth, mode of delivery and breastfeeding were not associated with viral response. Results trended across all methods in a direction consistent with benefits of breastfeeding for better viral response to early ART (Table 2, Table 3 and Table 4).

When examining markers of disease severity in infants pre-ART, median infant pre-treatment VL was higher in the ‘virologic failure’ group (189,250 copies/mL) compared to the ‘virologic success’ (11,909 copies/mL) group (*p* = 0.02) (Table 2). A similar pattern was also observed when pre-treatment VL was analyzed as a categorical variable, although not significant. Consistently, using the second method we found that lower pre-treatment VL (<1000 copies/mL) was associated with a higher probability of viral suppression <50 copies/mL compared to pre-treatment VL >100,000 copies/mL (HR = 3.00, 95% CI: 1.36–6.61), with similar findings for cut-off values of <400 copies/mL and TND (Table 3). For the third method, median infant pre-treatment VL was significantly higher in the ‘persistently high’ trajectory (290,807 copies/mL) than the other two groups (*p* = 0.0007) (Table 4). This was also observed when analyzed categorically, with a higher proportion of infants in the ‘persistently high’ trajectory (69.2%) having a pre-treatment VL ≥100,000 copies/mL. All of the infants in the ‘virologic failure’ group in Method 1 and 91% of the infants in the “persistently high’ group in Method 3 had an infant pre-treatment CD4 percentage ≥30% (Table 2). Pre-treatment CD4 was not associated with viral response by Method 2. Results were similar when using the imputed pre-treatment CD4 values.

When we examined maternal characteristics, we found in Method 3, that a larger proportion of infants who had no exposure to any maternal ART until delivery were in a better trajectory group [‘rapid decline’ (38.9%) or ‘slow decline’ (13.8%)] than those a ‘persistently high’ trajectory where no infants had exposure to maternal ART (*p* = 0.01) (Table 4). This association was also observed in Method 2, with a lower probability of VL <TND (HR = 0.46, 95% CI: 0.22–0.97) among those who received maternal ART during pregnancy. Results were in a similar direction for time to VL <50 and <400 copies (Table 3). A trend in this direction was also observed using clinical endpoints, with a greater proportion of infants in the ‘virologic success’ group (27.3%) born to mothers with no ART compared to those in the other groups (*p* = 0.06) (Table 2). To better understand this, we stratified those who received maternal ART during pregnancy into those with a maternal VL <1000 vs. ≥1000 copies/mL. We found that if mothers were on ART, having an elevated maternal VL ≥1000 copies/mL close to birth was associated with a less favorable infant viral response (Table 2, Table 3 and Table 4). In the full group of infants, higher maternal CD4 was associated with better infant viral response across all three methods.

## 4. Discussion

We used three methods to operationalize viral response in the context of early infant treatment and determine factors associated with viral response. As demonstrated in this analysis, the methods describe viral response in different ways. The clinical endpoint was derived from a protocol-defined endpoint which determined virologic success to be a VL <400 copies/mL by 24 weeks after ART initiation and a VL <50 copies/mL by 48 weeks of age, and no confirmed VL >50 copies/mL after suppression was attained. The remaining infants were further divided into two groups: ‘virologic rebound’ and ‘virologic failure’. Meanwhile, the time-dependent analysis considered the time taken to reach different thresholds of viral suppression for the first time (<400, <50, or TND). While this method can be useful to modulate the threshold of viral suppression, it completely ignores events (i.e., rebound) that take place once suppression is achieved. Finally, the LCGA analysis uses all data points over time to identify latent classes with qualitatively distinct trajectory patterns. Our results demonstrate that different information about virological response patterns can be ascertained, depending on the analytic method. Only using a single method to operationalize viral response, such as the time-dependent analysis, may result in an incomplete description of the response trajectory. While HIV trials have historically used a single method to assess virologic endpoints in HIV trials [9,10,11], it may be more comprehensive to present analyses of viral response using several methods.

In this study, factors associated with viral response were largely consistent across the three methods. Higher infant pre-treatment VL, particularly at a threshold above 100,000 copies/mL, was consistently associated with poorer viral response across all three methods. A study of 44 neonates starting ART in the first 28 days of life also found that very high baseline VL (>5 log10) strongly influenced time to VL suppression [12]. In contrast, a study in Botswana that initiated 40 infants on ART in the first week of life did not find any association between infant pre-treatment VL and VL suppression <40 copies at 12 weeks and 24 weeks [17].

We also observed that maternal CD4 independent of ART use was associated with better infant viral response. This finding is consistent with other studies which have shown associations between maternal health and disease progression in children living with HIV, and further emphasizes the importance of supporting optimal adherence for mothers on ART or initiating ART during pregnancy, and identifying new maternal infections during pregnancy [18,19,20].

While studies of children with HIV have found sex differences in viral response, we did not observe any associations between sex and viral response in our study [21,22]. The previously mentioned study of very early treated neonates in Botswana also did not find any association between sex and VL suppression <40 copies at 12 weeks and 24 weeks [17]. Furthermore, similar to the Botswana study, we did not observe any association between gestational age and viral response. In contrast, a study of intrauterine-infected infants in KwaZulu-Natal, South Africa found that infants born at a younger gestational age had lower rates of viral rebound [23]. For breastfeeding, we found a signal that breastfeeding may be associated with better viral response to early ART.

Studies of infants have reported the benefits of earlier ART initiation on long-term virologic control [3,4,5,6,7], but fewer studies have focused on infants who initiated ART very early in the neonatal period. Our data indicated that later ART initiation (8–14 days) may lead to less desirable viral outcomes compared to earlier ART initiation 0–48 h and >48 h–7days by the time-dependent method and the LCGA method. While there was a trend using the clinical endpoints, it was not significant. This is consistent with a longitudinal analysis of data from four cohorts in Italy, the UK, Thailand, and Spain, which assessed viral response using time-to-suppression methods as well as markers of lack of sustained viral control after suppression (e.g., failure, blips, and spikes) found that infants who initiated ART within 7 days were more likely to suppress earlier than those who started between 8–28 days (a wider window than in our study) by the time-to-event analysis [12]. In that study, the markers of lack of sustained viral control did not differ between the two age at ART start groups. Of note, our study did not focus on the viral reservoir or immunological outcomes, but benefits of earlier ART initiation on these outcomes has also been reported [24,25,26]. In addition, virologic response was poor overall; this study had planned an analytic treatment interruption study but the number of children meeting protocol-specified virologic and immunologic endpoints was too low to justify the interruption trial [8].

Two methods found higher infant pre-treatment CD4 percentage among infants who were categorized in the ‘virologic failure’ group by the clinical endpoint method or the ‘persistently high’ trajectory by the LCGA method. Unfortunately, infant CD4 was missing in a large proportion of the cohort (18%). We attempted to impute missing values but findings did not change. In contrast to our study, another study of infants starting ART within 6 months of life found pre-treatment CD4 percentage to be a predictor of faster time to suppression [27]. Using a mathematical model characterizing the kinetics of viral decay on ART among a subset of infants from our cohort, those with higher pre-treatment CD4 percentage had more rapid viral suppression [28]. However, that analysis was conducted only among the subset of infants who maintained a consistent decreasing trend toward suppression, suppressed VL below 20 copies/mL within 1 year, and had sufficient VL data.

We also found that infants with mothers who received any ART during pregnancy had worse viral outcomes compared to infants with mothers who did not receive any ART during pregnancy. Infants born to mothers who received any ART during pregnancy may have been infected earlier in pregnancy and exposed to virus for a longer duration in utero, or their mothers may not have adhered well to their own treatment or had drug resistance. Among those born to mothers on any ART during pregnancy, an elevated maternal VL was associated with a less favorable infant viral response. It is also possible that infants born to mothers who did not receive ART may have had other host factors (HIV-specific immunity or genetic factors) that led to better viral control [29,30]. A similar finding was reported in a study of intrauterine-infected infants in KwaZulu-Natal, South Africa, where a longer duration of maternal ART during pregnancy was found to predict infant viral rebound [23]. Further data, particularly on maternal and infant drug resistance, are needed to disentangle and better understand these pathways.

This study was strengthened by the rigorous collection of follow-up data from a trial protocol, and the narrow timeframe within which neonates initiated ART, allowing us to assess outcomes of very early treatment. However, our study had limitations. We selected a sequence of antiretroviral drug regimens considered optimal for treatment of neonates that were available at the time the study was done [8]. Adjunctive use of monoclonal antibodies and newer antiretrovirals, including integrase inhibitors, may help facilitate better outcomes. We had missing data, particularly for pre-treatment infant CD4, and maternal CD4 and VL closest to birth. Given HIV testing of infants at birth within 48 h, infant infection was assumed to occur intrauterine, but timing of maternal acquisition of infection was unknown. In addition, there were a number of additional factors that could have influenced viral response that were not measured in this study, such as adherence or other maternal health markers. Further, with all three methods, there is concern as to whether infrequent measurements of VL can truly reflect a dynamic pattern and whether an individual is suppressed over time.

## 5. Conclusions

We described viral response following very early treatment with ART in neonates using three different methods: clinical endpoints, time-dependent methods, and LCGA. We demonstrated similarities and differences in infant viral response assessed using these methods, and future studies should carefully consider these differences in decisions on how to operationalize viral response in their analyses. Finally, we found that factors associated with viral response, including infant pre-treatment VL, maternal VL, and maternal CD4 count, were largely consistent across the three methods.

## Figures and Tables

**Table 1:** Characteristics of 61 intrauterine HIV-infected neonates and their mothers enrolled at Rahima Moosa Mother and Child Hospital in Johannesburg, South Africa.

Characteristic	*n* = 61
Infant sex, *n* (%)	
Male	31 (50.8)
Female	30 (49.2)
Birth weight (g), Mean (SD)	2766 (629)
Birth weight (g), *n* (%)	
<2500	15 (24.6)
≥2500	46 (75.4)
Gestational age (weeks), *n* (%)	
≥37 (term)	51 (83.6)
<37 (pre-term)	10 (16.4)
Mode of delivery, *n* (%)	
Vaginal	46 (75.4)
Cesarean	15 (24.6)
Infant ever breastfed, *n* (%)	
Yes	46 (75.4)
No	15 (24.6)
Infant age at ART initiation, *n* (%)	
0–48 h	35 (57.4)
>48 h–7 days	18 (29.5)
8–14 days	8 (13.1)
Infant pre-treatment HIV RNA (copies/mL), Median (IQR)	19,400 (1720–240,810)
Infant pre-treatment viral load (copies/mL), *n* (%)	
<1000	13 (22.8)
1000–100,000	25 (43.9)
≥100,000	19 (33.3)
Infant pre-treatment CD4 count (cells/mm^3^), Mean (SD)	1990 (995)
Infant pre-treatment CD4 percentage (%), Mean (SD)	41.1 (13.2)
Infant pre-treatment CD4 percentage (%), *n* (%)	
<30	12 (24.0)
≥30	38 (76.0)
Mother’s age (years), Mean (SD)	28.8 (6.1)
Maternal ART during pregnancy, *n* (%)	
ART started before pregnancy and continued	10 (16.4)
ART started during pregnancy	40 (65.6)
No ART up until delivery	11 (18.0)
Maternal HIV RNA closest to birth (copies/mL), Median (IQR)	29,800 (1163–121,000)
Maternal HIV RNA closest to birth (copies/mL), *n* (%)	
<1000	14 (23.0)
≥1000	47 (77.1)
Maternal CD4 count closest to birth (cells/mm^3^), Mean (SD)	377 (264)
Maternal CD4 count closest to birth (cells/mm^3^), *n* (%)	
<350	36 (59.0)
≥350	25 (41.0)

Abbreviations: ART—antiretroviral therapy.

**Table 2:** Factors associated with viral response following clinically meaningful viral load endpoints (virologic success, virologic rebound, and virologic failure) by 72 weeks.

Characteristic	Virologic Success	Virologic Rebound	Virologic Failure	P1	P2	P3
	*n* = 33	*n* = 13	*n* = 15	Trend	S vs. R	S vs. F
Infant age at ART initiation (days), Mean (SD)	2.9 (2.8)	2.5 (2.9)	4.9 (4.4)	0.09	0.61	0.07
Infant age at ART initiation (days), Median (IQR)	1.0 (1.0–5.0)	1.0 (1.0–2.0)	4.0 (1.0–8.0)	0.43	0.59	0.26
Infant age at ART initiation, *n* (%)				0.24	0.48	0.26
0–48 h	19 (57.6)	10 (76.9)	6 (40.0)			
>48 h–7 days	11 (33.3)	2 (15.4)	5 (33.3)			
8–14 days	3 (9.1)	1 (7.7)	4 (26.7)			
Infant sex, *n* (%)				0.73	1.00	1.00
Male	16 (48.5)	7 (53.9)	8 (53.3)			
Female	17 (51.5)	6 (46.1)	7 (46.7)			
Birth weight (g), Mean (SD)	2759 (607)	2754 (757)	2795 (601)	0.87	0.98	0.85
Birth weight (g), *n* (%)				0.58	1.00	0.73
<2500	9 (27.3)	3 (23.1)	3 (20.0)			
≥2500	24 (72.7)	10 (76.9)	12 (80.0)			
Gestational age (weeks), *n* (%)				0.69	1.00	0.69
<37 (pre-term)	5 (15.1)	2 (15.4)	3 (20.0)			
≥37 (term)	28 (84.9)	11 (84.6)	12 (80.0)			
Mode of delivery, *n* (%)				0.58	1.00	0.73
Vaginal	24 (72.7)	10 (76.9)	12 (80.0)			
Cesarean	9 (27.3)	3 (23.1)	3 (20.0)			
Infant ever breastfed, *n* (%)				0.22	0.43	0.28
Yes	27 (81.8)	9 (69.2)	10 (66.7)			
No	6 (18.2)	4 (30.8)	5 (33.3)			
Infant pre-treatment viral load (copies/mL), Median (IQR)	11,909 (1124–48,905)	9849 (436–136,045)	189,250 (30,368–391,000)	0.07	0.73	0.02
Infant pre-treatment viral load (copies/mL), *n* (%)				0.12	0.36	0.10
<1000	7 (23.3)	5 (38.5)	1 (7.1)			
1000–100,000	16 (53.3)	4 (30.8)	5 (35.7)			
≥100,000	7 (23.3)	4 (30.8)	8 (57.1)			
Infant pre-treatment CD4 count (cells/mm^3^), Mean (SD)	2049 (1158)	1632 (698)	2233 (777)	0.85	0.26	0.63
Infant pre-treatment CD4 percentage (%), Mean (SD)	40.1 (13.6)	38.9 (14.1)	45.9 (10.9)	0.30	0.79	0.22
Infant pre-treatment CD4 percentage (%), *n* (%)				0.04	0.72	0.04
<30	9 (33.3)	3 (25.0)	0			
≥30	18 (66.7)	9 (75.0)	11 (100.0)			
Maternal ART during pregnancy, *n* (%)				0.06	0.24	0.14
Any ART	24 (72.7)	12 (92.3)	14 (93.3)			
No ART up until delivery	9 (27.3)	1 (7.7)	1 (6.7)			
Among those on any ART during pregnancy, maternal viral load closest to birth (copies/mL), *n* (%)				0.09	0.15	0.08
<1000	7 (29.2)	6 (50.0)	0 (0.0)			
≥1000	17 (70.8)	6 (50.0)	14 (100.0)			
Maternal CD4 count closest to birth (cells/mm^3^), Mean (SD)	399 (271)	391 (328)	317 (178)	0.35	NA	NA
Maternal CD4 count closest to birth (cells/mm^3^), *n* (%)				0.02	0.20	0.03
<350	15 (45.5)	9 (69.2)	12 (80.0)			
≥350	18 (54.5)	4 (30.8)	3 (20.0)			

**Table 3:** Factors associated with time to any HIV-1 RNA viral load <400 copies/mL, <50 copies/mL, or target not detected (TND) by 72 weeks; presented are hazard ratios (HR) from Cox proportional hazards regression models; HR >1.0 indicates parameter is protective and reflects “better viral suppression”.

Factor	Category	<400	<50	<TND
Infant age at ART initiation (days)	Continuous	0.96 (0.89, 1.04)	0.92 (0.84, 1.00)	0.92 (0.83, 1.01)
Infant age at ART initiation	0–48 h	-	2.46 (0.94, 6.45)	1.12 (0.36, 3.50)
	>48 h–7 days	-	2.18 (0.89, 6.03)	0.71 (0.18, 2.84)
	8–14 days	-	Ref.	Ref.
Sex	Male	0.78 (0.46, 1.31)	0.84 (0.48, 1.47)	0.66 (0.35, 1.24)
	Female	Ref.	Ref.	Ref.
Birth weight (grams)	<2500	Ref.	Ref.	Ref.
	≥2500	0.82 (0.45, 1.51)	0.78 (0.41, 1.48)	0.68 (0.35, 1.35)
Gestational age (weeks)	<37 (pre-term)	Ref.	Ref.	Ref.
	≥37 (term)	1.02 (0.50, 2.08)	1.07 (0.50, 2.28)	0.88 (0.39, 2.00)
Mode of delivery	Vaginal	0.77 (0.42, 1.41)	0.92 (0.48, 1.76)	0.78 (0.39, 1.56)
	Cesarean	Ref.	Ref.	Ref.
Infant breastfeeding	Ever breastfed	0.86 (0.47, 1.58)	1.20 (0.61, 2.35)	1.01 (0.49, 2.08)
	Not breastfed	Ref.	Ref.	Ref.
Infant pre-treatment HIV-1 RNA viral load (copies/mL)	<1000	5.93 (2.71, 12.95)	3.00 (1.36, 6.61)	3.44 (1.42, 8.34)
	1000–100,000	2.20 (1.13, 4.26)	1.70 (0.84, 3.43)	1.73 (0.76, 3.92)
	≥100,000	Ref.	Ref.	Ref.
Infant CD4 percentage (%)	<30	Ref.	Ref.	Ref.
	>30	1.25 (0.64, 2.47)	0.80 (0.40, 1.60)	1.12 (0.50, 2.52)
Maternal ART during pregnancy	Any ART	0.59 (0.30, 1.15)	0.58 (0.29, 1.17)	0.46 (0.22, 0.97)
	No ART up until delivery	Ref.	Ref.	Ref.
Among those on any ART during pregnancy, maternal viral load closest to birth (copies/mL)	<1000	3.12 (1.59, 6.14)	3.57 (1.76, 7.23)	1.64 (0.75, 3.60)
	≥1000	Ref.	Ref.	Ref.
Maternal CD4 count closest to birth (cells/mm^3^)	<350	Ref.	Ref.	Ref.
	≥350	1.46 (0.85, 2.52)	2.31 (1.29, 4.12)	1.81 (0.97, 3.39)

**Table 4:** Factors associated with viral response through 72 weeks following trajectory membership groups determined by latent class growth analysis.

Characteristic	Group = 1 Rapid Decline	Group = 2 Slow Decline	Group = 3 Persistently High	P1	P2	P3
	*n* = 18 (29.5%)	*n* = 29 (47.5%)	*n* = 14 (23.0%)	Trend	1 vs. 2	1 vs. 3
Infant age at ART initiation (days), Mean (SD)	2.7 (2.4)	2.4 (2.7)	6.0 (4.4)	0.008	0.78	0.01
Infant age at ART initiation (days), Median (IQR)	1.0 (1.0–5.0)	1.0 (1.0–4.0)	6.0 (1.0–9.0)	0.11	0.44	0.04
Infant age at ART initiation, *n* (%)				0.03	0.44	0.09
0–48 h	10 (55.6)	21 (72.4)	4 (28.6)			
>48 h–7 days	7 (38.9)	6 (20.7)	5 (35.7)			
8–14 days	1 (5.6)	2 (6.9)	5 (35.7)			
Infant sex, *n* (%)				0.47	0.19	0.53
Male	7 (38.9)	17 (58.6)	7 (50.0)			
Female	11 (61.1)	12 (41.4)	7 (50.0)			
Birth weight (g), Mean (SD)	2814 (645)	2798 (610)	2639 (677)	0.46	0.93	0.46
Birth weight (g), *n* (%)				0.68	1.0	1.0
<2500	5 (27.8)	7 (24.1)	3 (21.4)			
≥2500	13 (72.2)	22 (75.9)	11 (78.6)			
Gestational age (weeks), *n* (%)				0.87	0.40	1.0
<37 (pre-term)	4 (22.2)	3 (1.3)	3 (21.4)			
≥37 (term)	14 (77.8)	26 (89.7)	11 (78.6)			
Mode of delivery, *n* (%)				0.41	1.0	0.43
Vaginal	13 (72.2)	21 (72.4)	12 (85.7)			
Cesarean	5 (27.8)	8 (27.6)	2 (14.3)			
Infant ever breastfed, *n* (%)				0.22	0.72	0.25
Yes	15 (83.3)	22 (75.9)	9 (64.3)			
No	3 (16.7)	7 (24.1)	5 (35.7)			
Infant pre-treatment viral load (copies/mL), Median (IQR)	9070	11,600	290,807	0.0007	0.56	0.0002
	(525–43,150)	(901–136,045)	(43,300–1,660,225)			
Infant pre-treatment viral load (copies/mL), *n* (%)				0.02	0.47	0.005
<1000	4 (26.7)	9 (31.0)	0 (0.0)			
1000–100,000	9 (60.0)	12 (41.4)	4 (30.8)			
≥100,000	2 (13.3)	8 (27.6)	9 (69.2)			
Infant pre-treatment CD4 count (cells/mm^3^), Mean (SD)	2619 (1094)	1650 (844)	1962 (864)	0.07	0.004	0.12
Infant pre-treatment CD4 percentage (%), Mean (SD)	49.4 (10.9)	36.6 (13.0)	40.8 (11.9)	0.07	0.003	0.07
Infant pre-treatment CD4 percentage (%), *n* (%)				0.03	0.06	1.0
<30	1 (7.1)	10 (40.0)	1 (9.1)			
≥30	13 (92.9)	15 (60.0)	10 (90.9)			
Maternal ART during pregnancy, *n* (%)				0.01	0.08	0.01
Any ART	11 (61.1)	25 (86.2)	14 (100.0)			
No ART up until delivery	7 (38.9)	4 (13.8)	0 (0.0)			
Among those on any ART during pregnancy, maternal viral load closest to birth (copies/mL), *n* (%)				0.03	1.0	0.05
<1000	4 (36.4)	9 (36.0)	0 (0.0)			
≥1000	7 (63.6)	16 (64.0)	14 (100.0)			
Maternal CD4 count closest to birth (cells/mm^3^), Mean (SD)	460 (250)	367 (287)	291 (209)	0.07	0.26	0.05
Maternal CD4 count closest to birth (cells/mm^3^), *n* (%)				0.005	0.006	0.004
<350	5 (27.8)	20 (69.0)	11 (78.)			
≥350	13 (72.2)	9 (31.0)	3 (21.4)			

## Data Availability

The data presented in this study are available on request from the corresponding author. The data are not publicly available due to privacy restrictions.

## Supplementary Materials

The following are available online at https://www.mdpi.com/article/10.3390/jcm10102074/s1, Table S1: Summary of methods used to define viral response through 72 weeks, Table S2: Bayesian Information Criterion (BIC) used to select groups from latent class growth analysis (LCGA).
