# Supplementary material for: Virologic Response to Very Early HIV Treatment in Neonates
Source: J Clin Med. 2021 May 12;10(10):2074. doi: 10.3390/jcm10102074 (PMC8151270; doi:10.3390/jcm10102074)
Supplement: Supplementary file 1 [file jcm-10-02074-s001.zip › jcm-1183070-supplementary.pdf]

Supplementary Table 1: Summary of methods used to define viral response through 72 weeks

Description	Definition
**Method 1:** Clinically-meaningful viral endpoints	Virologic success: VL <400 copies/mL by 24 weeks after ART initiation and a VL <50 copies/mL by 48 weeks of age, and no confirmed VL (i.e. two consecutive measurements) >50 copies/mL after suppression was attained through 72 weeks.<br>Virologic rebound: VL <400 copies/mL by 24 weeks after ART initiation, a VL <50 copies/mL by 48 weeks of age, and confirmed VL >50 copies/mL after suppression was attained through 72 weeks.<br>Viral failure: never having achieved VL <400 copies/mL by 48 weeks of age
**Method 2:**<br>Time to viral suppression	Time to any VL <400 copies/mL, <50 copies/mL, or target not detected (TND) by 72 weeks
**Method 3:** Latent class growth analysis (LCGA)	Fit latent trajectories on log10-transformed VLs (copies/mL) over time and selected the best fitting model with number of latent groups using the Bayesian Information Criterion (BIC), as well as the group membership posterior probability. Selected final model based on 1) the inflection point at which the ΔBIC leveled off and 2) clinical meaning of each latent group.

Supplementary Table 2: Bayesian Information Criterion (BIC) used to select groups from latent class growth analysis (LCGA)

Number of groups	BIC (*n* = 764)	Null model	ΔBIC	BIC (*n* = 61)	Null model	ΔBIC
1	-1516.19			-1511.14		
2	-1358.05	1	158.14	-1347.94	1	163.2
3	-1289.00	2	69.05	-1273.84	2	74.1
4	-1276.87	3	12.13	-1256.65	3	17.19
5	-1268.73	4	8.14	-1243.45	4	13.2

BIC = Bayesian Information Criterion

ΔBIC: BIC change between a more complex model and null model.
